# Severe refractory autoimmune hemolytic anemia with five-year complete hematologic response to third course of treatment with rituximab: a case report

**DOI:** 10.1186/1752-1947-8-175

**Published:** 2014-06-02

**Authors:** Kathleen Abadie, Kristen M Hege

**Affiliations:** 1 Division of Hematology/Oncology, University of California, 400 Parnassus Ave, Ste 502, San Francisco, CA 94143-0324, USA; 2 Department of Chemical and Biomolecular Engineering, Rice University, 6100 Main Street, Houston, TX 77005, USA

**Keywords:** Autoimmune hemolytic anemia, Rituximab, IgG subclass, Complement inhibitory proteins

## Abstract

**Introduction:**

Rituximab is an emerging treatment for autoimmune hemolytic anemia. We report the case of a patient with a five-year complete hematologic response to a third course of treatment with rituximab. Cases of response to rituximab re-treatments have been reported, but none to our knowledge that failed multiple prior treatments and achieved as durable a response.

**Case presentation:**

A 45-year-old Hispanic man presented at age 26 with darkening urine and cold intolerance. His blood tests revealed elevated lactic dehydrogenase and bilirubin, a hemoglobin level of 7.4g/dL, and a positive Coombs test for complement C3 and immunoglobulin G antibody. A diagnosis of autoimmune hemolytic anemia was made. After failing multiple therapies including prednisone, splenectomy, immunoglobulin, cyclosporine, danocrine and azathioprine, our patient was treated with a four-week course of rituximab at a dose of 375mg/m^2^ weekly, 10 years following initial presentation. He achieved a rapid and complete hematologic response that lasted 25 months. Re-treatment with the same course of rituximab prompted a second response that lasted 18 months. A third re-treatment has achieved an ongoing five-year complete hematologic response.

**Conclusions:**

This is an unusual case of a durable five-year remission of autoimmune hemolytic anemia with rituximab re-treatment following relapse after two prior courses of rituximab and despite the persistence of immunoglobulin G and complement-coated red blood cells. No mechanistic explanations for improved response to rituximab re-treatment in autoimmune hemolytic anemia have been reported in the literature. Future studies of rituximab or other B cell-targeting antibodies in the treatment of autoimmune hemolytic anemia should explore autoantibody immunoglobulin G subclass switching and alterations in complement inhibitory proteins on red blood cell membranes as potential correlates of hematologic response.

## Introduction

Autoimmune hemolytic anemia (AIHA) is characterized by B cell production of autoantibodies that bind to red blood cell surface antigens and initiate red blood cell destruction. Treatments aim to reduce autoantibody production or immune-mediated clearance of opsonized red blood cells and include glucocorticoids and other immunosuppressive therapies, pooled gamma globulins, cytotoxic agents, monoclonal antibodies, and splenectomy. Rituximab, an immunoglobulin G1 (IgG1) kappa chimeric monoclonal antibody directed against the CD20 antigen expressed on B cells, has established efficacy in the treatment of refractory AIHA. A 2009 Belgian retrospective study yielded a 79 percent hematologic response rate in 53 subjects with refractory AIHA treated with rituximab [1], and a 2009 Spanish retrospective study similarly demonstrated a 77 percent response rate in 36 subjects with AIHA [2]. Achievement of a negative direct antiglobulin (Coombs) test following administration of rituximab has been shown to be a predictor of more durable responses [2]. The Belgian study noted some cases of re-treatment responses to rituximab that lasted longer than the initial treatment [1].

Here we present the case of a patient with an ongoing five-year complete hematologic response to a third course of rituximab for the treatment of multiply relapsed AIHA following two prior rituximab responses of shorter duration. Our patient’s hemoglobin and hematocrit levels remain in the normal range despite a persistently positive Coombs test. This unusual case supports the utility of rituximab re-treatment and prompts further investigation into the mechanism of action of rituximab and its effect on the pathogenicity of red blood cell autoantibody and complement-mediated hemolysis.

## Case presentation

Our patient is a 45-year-old Hispanic man first diagnosed with AIHA at age 26 in January 1995. He initially presented after noticing darkening of his urine and cold intolerance in the month preceding his diagnosis. Laboratory tests revealed elevated lactic dehydrogenase (LDH) and bilirubin, a hemoglobin level of 7.4g/dL, and a Coombs test positive for complement (C3) and immunoglobulin G (IgG) antibody. Further evaluation ruled out an underlying lymphoma, other malignancy, or an associated systemic autoimmune disorder, and a diagnosis of AIHA was made. His anemia initially responded to prednisone at a dose of 80mg daily, but multiple attempts to taper and discontinue prednisone resulted in recurrent hemolysis and anemia. Additional therapies included pulse dexamethasone treatment from January to April 1996 and a splenectomy in August 1996. Hemolysis and anemia persisted following the splenectomy, and he was subsequently treated with various combinations of prednisone, intravenous immunoglobulin, danocrine, cyclosporine, and azathioprine. His treatment course was complicated by cryptococcal meningitis in April of 1997 and refractory cutaneous warts secondary to chronic immunosuppression. In addition, he had an acute myocardial infarction in October 2001 at the age of 32.

**Figure 1:**
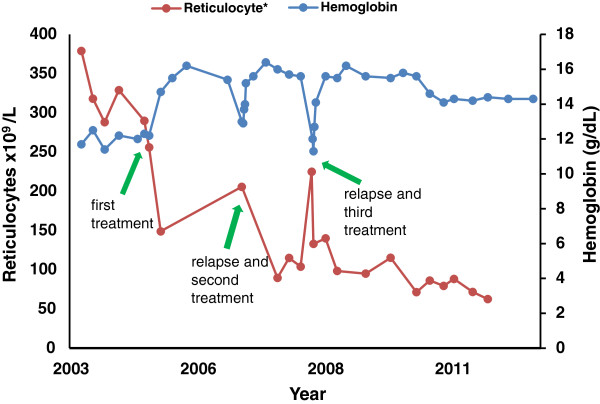
**Hemoglobin levels and reticulocyte counts from 2003 to 2013.** *Method of measuring reticulocytes changed in December, 2007.

Following initial case reports of hematologic response in refractory AIHA treated with rituximab [3,4], our patient received his first treatment with a four-week course of rituximab at a dose of 375mg/m^2^ weekly in February, 2005. Pretreatment laboratory tests showed a hemoglobin level of 12.3g/dL, reticulocyte count of 290×10^9^/L, and persistent positive Coombs test with IgG 3+ and C3 1+. He achieved a rapid and complete hematologic response with normalization of his hemoglobin and reticulocyte count for the first time in his history. By June 2005, his hemoglobin and reticulocyte count were in the normal range at 14.7g/dL and 149×10^9^/L, respectively. Chronic immune suppressive therapy with prednisone, danocrine and azathioprine was tapered and stopped over the next four months. This first response to rituximab lasted 25 months. Recurrent hemolysis developed in March 2007, manifested by a hemoglobin count of 13.0g/dL and reticulocyte count of 206×10^9^/L, and prompted re-treatment with another four-week course of rituximab. Our patient achieved a second complete hematologic response with a rise in hemoglobin level to 16.4g/dL and decrease in reticulocyte count to 105×10^9^/L. Hemolysis again recurred in September 2008 with a drop in his hemoglobin level to 11.3g/dL after an 18-month duration of response. He was re-treated with a third four-week course of rituximab at the same dose and schedule. Since this last course of rituximab he has maintained an ongoing five-year complete hematologic response with hemoglobin levels within the normal range through September 2013.This third hematologic response (ongoing at five years) is more than twice the duration of either of his previous responses (25 and 18 months, respectively) and persists despite the continued presence of IgG antibodies and complement on his red blood cells, detected by a persistently positive Coombs test (IgG 4+, C3 1+). He has been off all immunosuppressive therapy other than the three courses of rituximab since 2005. Absolute lymphocyte count and B and T cells subsets in his blood, as measured by flow cytometry, are within normal limits. Maintenance fluconazole therapy for cryptococcal meningitis was stopped in 2005 without recurrence of meningitis, and his refractory cutaneous warts have completely resolved without specific therapy. He has suffered no additional cardiac ischemic episodes. A longitudinal summary of his hemoglobin and reticulocyte counts as well as rituximab administration is presented in Figure 1.

## Discussion

This case report describes a durable five-year complete hematologic response to rituximab in a subject with refractory AIHA despite relapse following two prior rituximab treatments and the persistence of a positive Coombs test. This case raises two related questions. First, why are the autoantibodies and complement persistently detected on the patient’s red blood cells no longer causing hemolysis, and what mechanistic role might rituximab have played in this transition? Second, why did a third course of treatment with rituximab result in a lasting hematologic remission, when the same treatment regimen resulted in much shorter response durations on two previous occasions? Here we conduct an exploration of these questions based on literature review and retrospective analysis of this case.

Several factors influence the pathogenicity of red blood cell-bound autoantibodies and associated induction of hemolysis. These factors include antibody characteristics involving class, subclass, specificity, thermal amplitude, complement-activating efficiency, affinity and amount of galactose on the (crystallizable fragment (Fc) carbohydrate), quantity of cell-bound antibodies, and activity of the complement-mediated and phagocytic clearance systems [5]. Of these factors, rituximab is most likely to affect red blood cell autoantibody characteristics, due to its mechanism of action of selective B cell depletion [6]. One possible explanation for the loss of autoantibody pathogenicity is a change in IgG subclass. The human IgG subclasses IgG1, IgG2, IgG3, and IgG4 have identical variable regions but differ in the heavy chain. The pathogenic potential of autoantibodies is directly related to the binding properties of the heavy chain Fc region, and variation in pathogenicity and hemolysis induction between subclasses has been shown in mice [7]. In humans, IgG4 autoantibodies are known to be poorly pathogenic, lacking the ability to activate complement and trigger macrophage phagocytosis. IgG2 autoantibodies activate complement poorly and only react with macrophages if the Fc receptor is of a particular allotype [5]. It is therefore plausible that a switch from a highly pathogenic subclass, IgG1 or IgG3, to a poorly pathogenic subclass, IgG2 or IgG4, could be responsible for the termination of our patient’s hemolysis, despite persistence of red blood cell-bound autoantibodies. Further exploration is warranted to determine if rituximab response in AIHA is associated with IgG subclass switching. In future studies, the relative levels of different IgG subclasses on red blood cells before and after rituximab treatment might be tested by nephelometry or another known method.

Several researchers have suggested the immune complex decoy hypothesis as an important component of the mechanism of action of rituximab and an explanation for its rapid effect in inhibiting hemolysis [8]. The complex decoy hypothesis asserts that the immune complexes formed by rituximab bound to B cells serve as alternative substrates for both circulating monocytes and fixed tissue macrophages, thereby distracting these effector cells from the destruction of IgG and C3-opsonized red blood cells [8]. Inhibition of hemolysis associated with this complex decoy hypothesis is postulated to be transitory in nature, and though it may explain our patient’s rapid response to rituximab after each treatment, it cannot explain the ongoing five-year hematologic response. This hematologic remission persists in the presence of normal circulating B and T lymphocyte counts (absolute lymphocyte count 1.7×10^9^/L, CD3+ T cells 935×10^6^/L, CD19+ B cells 629×10^6^/L) and long after the clearance of rituximab and rituximab-bound B cells from his system.

Complement inhibitory proteins such as CD55 and CD59 that protect from uncontrolled complement-mediated red blood cell lysis may also play a role in our patient’s hematologic remissions. CD55 and CD59 are glycosylphosphatidylinositol-anchored, type I cell surface proteins that inhibit formation of the C3 convertases and prevent the terminal polymerization of the membrane attack complex, respectively [9]. A 2009 study by Barros *et al*. demonstrated low expression of CD59 in warm autoimmune hemolytic anemia, while CD55 expression was unchanged between AIHA patients and healthy individuals [10]. Barros *et al.* conclude that complement inhibitory proteins may play an important role in protecting red blood cells from destruction by complement [10]. This prompts consideration of the possibility that upregulation of complement inhibitory proteins, such as CD55 and CD59, from low to normal levels might represent an additional potential factor in the mechanism of action of rituximab in treating AIHA. As these complement inhibitory proteins are involved in regulation of B cell destruction by rituximab, interplay between the inhibitory proteins, rituximab, and the C3-opsonized red blood cells might contribute to the hematologic response observed with rituximab treatment in AIHA. A 2001 study by Weng and Levy found no change in CD55 or CD59 levels measured by flow cytometry in patients with non-Hodgkin lymphoma following rituximab treatment [9], but this topic warrants further exploration, specifically in AIHA, by testing CD55 and CD59 levels on red blood cells by flow cytometry before and after rituximab treatment.

It remains uncertain why our patient developed recurrent hemolysis after two prior rituximab treatments, with response durations of 25 and 18 months, respectively, but is experiencing a lasting response, ongoing at five years, to the third course of treatment. Previous cases of increased response durations following retreatment of AIHA with rituximab have been observed [1], but to the best of our knowledge, five-year hematologic remissions following multiple prior relapses have not previously been reported.

## Conclusions

This report describes an unusual case of a durable five-year remission of AIHA with rituximab retreatment following relapse after two prior courses of rituximab and despite the persistence of IgG and complement-coated red blood cells. No mechanistic explanations for improved response to rituximab retreatment in AIHA have been reported in the literature. Future studies of rituximab or other B cell-targeting antibodies in the treatment of AIHA should explore autoantibody IgG subclass switching and alterations in complement inhibitory proteins on red blood cell membranes as potential correlates of hematologic response.

## Consent

Written informed consent was obtained from the patient for publication of this case report and any accompanying images. A copy of the written consent is available for review by the Editor-in-Chief of this journal.

## Competing interests

The authors declare that they have no competing interests.

## Authors’ contributions

KH treated the patient and contributed to the writing of the manuscript. KA wrote the manuscript. Both authors approved the final version of the manuscript.

## Authors’ information

Kathleen Abadie is a student at Rice University who worked with Dr. Hege as a summer intern in 2013. Kristen Hege is a part-time UCSF faculty member who is also employed by Celgene Corporation.
